# FAPI-PET/CT guided radiotherapy for patients with esophageal cancer

**DOI:** 10.1186/s13014-025-02606-x

**Published:** 2025-02-28

**Authors:** Kai Kröger, Niklas Benedikt Pepper, David Ventura, Fabian M. Troschel, Philipp Backhaus, Kambiz Rahbar, Bernhard Glasbrenner, Matthias Brüwer, Andreas Pascher, Michael Schäfers, Hans Theodor Eich, Wolfgang Roll

**Affiliations:** 1 Department of Radiation Oncology, University Hospital Muenster, Muenster, Germany; 2 Department of Nuclear Medicine, University Hospital Muenster, Muenster, Germany; 3 European Institute for Molecular Imaging, University of Münster, Muenster, Germany; 4 Department of Medicine B, Gastroenterology, St. Franziskus-Hospital Muenster, Muenster, Germany; 5 Department of General and Visceral Surgery, Gastroenterology, St. Franziskus-Hospital Muenster, Muenster, Germany; 6 Department of General, Visceral and Transplantation Surgery, University Hospital Muenster, Muenster, Germany

**Keywords:** FAPI, Esophageal cancer, PET, Radiotherapy

## Abstract

**Background:**

Cancer associated fibroblasts have become a target of interest in different malignancies for positron emission tomography (PET) imaging, using positron emitter labelled fibroblast activation protein inhibitors (FAPI). New data underline the advanced imaging properties of FAPI-PET/CT for the staging of esophageal cancer compared to standard imaging. Potential benefits of FAPI-PET/CT in radiation therapy planning are the subject of this investigation.

**Methods:**

Ten patients with newly diagnosed esophageal cancer treated with radiochemotherapy (RCT) were retrospectively analyzed. All patients underwent [68Ga]OncoFAP-PET/CT in treatment position to facilitate radiation treatment planning. Six patients received neoadjuvant RCT as part of a trimodal therapy and four patients underwent definitive RCT. In five cases, restaging after initial treatment was performed with FAPI-PET/CT.

**Results:**

[68Ga]OncoFAP-PET/CT based imaging showed a high correlation with the endoscopic staging for initial imaging. In three cases, new sites of disease were unmasked, not visible in CT- and endosonographic staging. [68Ga]OncoFAP-PET/CT based RT delineation offered good definition of clinical target volumes, especially in retro-/paracardial areas and the gastroesophageal junction.

**Conclusion:**

[68Ga]OncoFAP-PET/CT may aid and improve radiation treatment planning for patients with esophageal cancer.

## Background

The incidence and mortality of esophageal cancer depend on the histological subtype and show high regional differences [1]. Squamous cell carcinoma (SCC) and adenocarcinoma are the most frequent histological subtypes. Globally, SCC is more prevalent, oftentimes being associated with behavioral risk factors and particularly frequent in low-income countries [1]. SCC tends to have a more aggressive course and poorer prognosis, leading to increased mortality rates compared to adenocarcinoma [2].

In advanced stages, optimal treatment usually requires a multimodal approach, including surgery and radiochemotherapy (RCT). If resection is not possible due to location (e.g., cervical esophagus) or patient characteristics (e.g., co-morbidities), definitive RCT is the standard of care [2, 3].

Pre-treatment workup staging includes upper gastrointestinal endoscopy with tumor site biopsy and endoscopic ultrasound (EUS), contrast enhanced computed tomography (CT) of the thorax and abdomen/pelvis and, if available, [18F]fluorodeoxyglucose positron emission tomography [18F]FDG-PET) [4]. For radiation treatment planning, precise definition of the target volumes is of utmost importance. However, the accurate identification of the tumor extension may be challenging due to limited contrast between the tumor and surrounding tissue. Transferring endoscopically obtained measurements of tumor spread to planning CT scans can be challenging. These difficulties play a key role especially in non-obstructing tumors that may not be visible on contrast-enhanced CT. Molecular imaging with [18F]FDG-PET, which visualizes increased glucose utilization, also faces some tracer-specific drawbacks:

- uptake in infection/inflammation; e.g. esophagitis in reflux disease.
- small tumors and lymph-node metastases can be false negative.
- uptake in surrounding tissue, especially myocardium and stomach can hamper target volume delineation for radiotherapy (RT).

Fibroblast activation protein (FAP) is an antigen expressed on cancer associated fibroblasts as part of the tumor microenvironment and on the surface of cancer cells in a variety of malignant neoplasms [5]. FAP inhibitors (FAPI) can be combined with ß-emitters such as [18F] or [68Ga] to be used in PET for diagnostic purposes and staging across different types of cancers [6]. Many different FAPI-PET compounds have been used and previously published in different clinical scenarios [6]. First results in esophageal cancer hint towards.

- improved tumor detection rates for FAPI-PET compared to [18F]FDG-PET/CT [7].
- a potential benefit for RT planning with significant differences in gross tumor volume (GTV) delineation [8].
- a potential prognostic value of FAPI-PET parameters to predict response to definite RCT [9, 10].

However, most research approaches mainly included SCC (with only a minority of adenocarcinoma due to reduced local prevalence [7, 9, 10]). Many previous studies focus on patients with definitive RCT [9, 10]. Emerging data suggest a potential role of FAPI-PET in assessing treatment response after neoadjuvant therapy [11].

In this retrospective study we present evidence of FAPI-PET-based RT planning in a real-world collective of esophageal cancer patients, including both SCC and adenocarcinoma as well as different radiation oncology treatment strategies. We also show first results on FAPI-PET response assessment after (neoadjuvant) RCT.

## Methods

### Patient selection

We retrospectively analyzed data from ten patients with a first diagnosis of esophageal cancer who underwent RCT with curative intent at our institution between January 2022 and January 2023. After initial consultation with the radiation oncologist and obtaining informed consent, all patients received a FAPI-PET/CT in the radiotherapy treatment position on an individual clinical basis, with the compound [68Ga]Ga-OncoFAP-DOTAGA ([68Ga]OncoFAP).

### [68Ga]OncoFAP-PET

[68Ga]OncoFAP-PET/CT scans were conducted 60 min after injection of a median of 168 MBq [68Ga]Ga-OncoFAP-DOTAGA (range: 106–226MBq). Precursor was provided by Philochem AG, Otelfingen, Switzerland. Imaging was performed on a PET/CT scanner (mCT, Siemens Healthineers, Munich, Germany). To ensure an optimal positioning of the patient the procedure was accompanied by a physician and a dosimetrist of the department of radiation oncology. The simultaneously acquired CT imaging consisted of a full body contrast enhanced CT. Notably, patients did not need to fast before the examination (as opposed to FDG-PET/CT). A nuclear medicine physician and radiation oncologist assessed PET imaging. Quantitative uptake measurements of active areas were obtained by calculation of maximal standard uptake value (SUVmax) and the SUVpeak (maximal standard uptake value in a 1cm^3^ volume of interest).

### Radiation therapy planning and image analysis

**Fig. 1:**
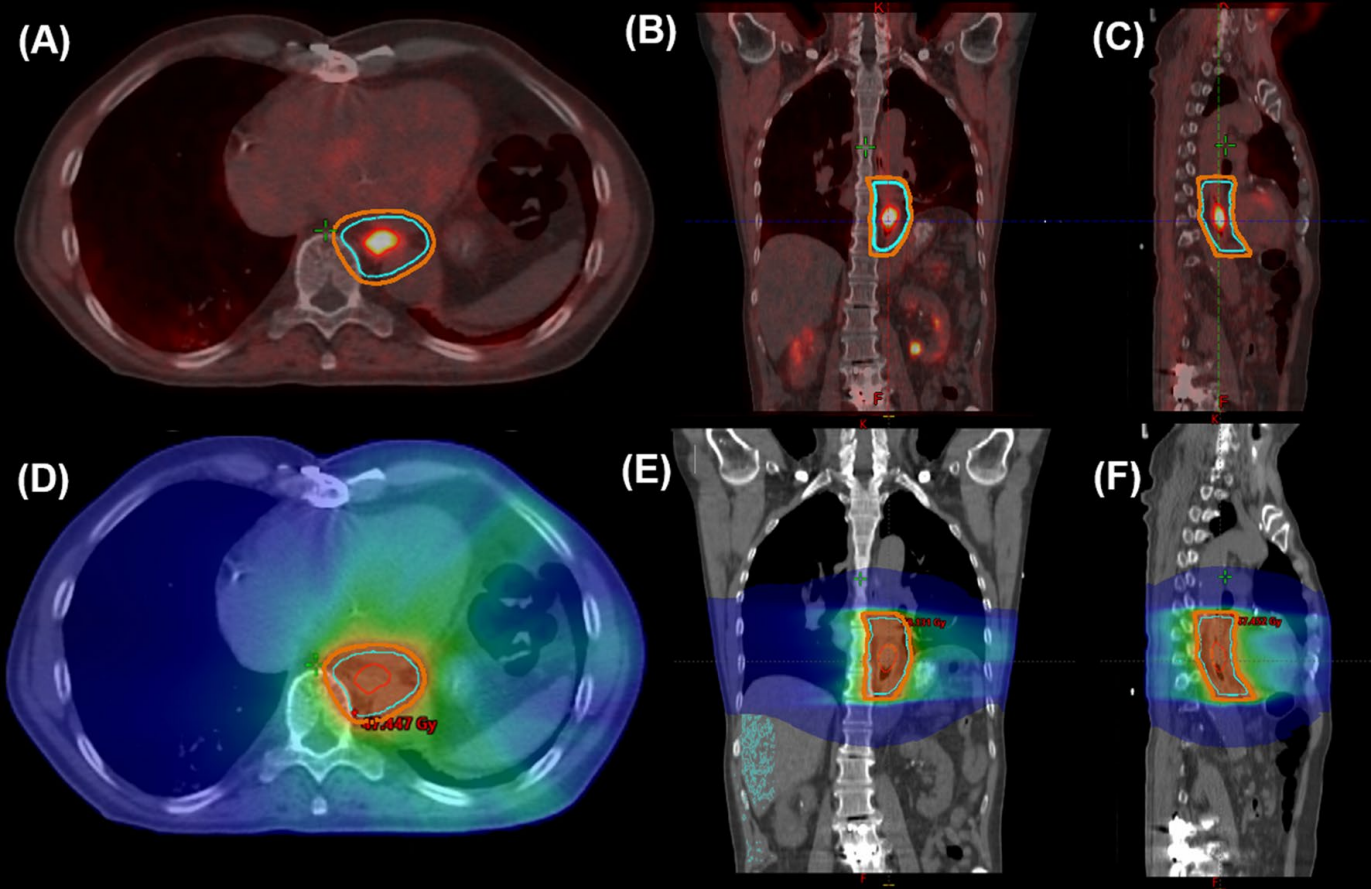
Exemplary target volume definition (**A**–**C**) for [68Ga]OncoFAP-PET/CT based neoadjuvant treatment in a patient is esophageal SCC. As part of target volume delineation, the [68Ga]OncoFAP-based GTVp_PET + CT (including the whole esophagus on the level of PET-activity) is depicted by the red outline, the resulting CTV by the cyan structure and the PTV in orange. Note the superb visibility of the PET-positive lesion directly adjacent to the heart. Additionally, the RT dose distribution is illustrated (**D**–**F**)

Delineation of target volumes and organs at risk (OAR) for RT was performed by a board-certified radiation oncologist after image import into the planning software Varian Eclipse 11.0 (provided by Varian Medical Systems, Palo Alto, CA, USA). Treatment planning was completed according to the current guidelines and the ICRU-Report 83 for treatment volume definition and dose prescription for intensity-modulated radiotherapy (IMRT) [12, 13]. Primary tumor and metastases visible in [68Ga]OncoFAP-PET imaging and contrast enhanced CT were used for the delineation of gross tumor volume (GTV). A lesion-based threshold of 40% was used to assess the PET-positive GTV. To further evaluate volumetric changes regarding the target volumes, additional structures were defined for study purposes: GTVp_CT and GTVn_CT, defined as (primary) tumor and nodal GTV using only information gained by CT and endoscopy, and GTVp_CT + PET and GTVn_CT + PET, defined as GTV also incorporating information gained by [68Ga]OncoFAP-PET/CT. Of note, the whole esophagus was contoured at the level of the PET-positive lesion for GTVp, not only the lesion itself. The PET-based planning process in a patient receiving neoadjuvant RCT (patient 10) is exemplarily illustrated in Fig. 1.

The primary clinical target volume (CTVp) around the corresponding GTVp was defined with a craniocaudal expansion of 4 cm and 0.5-1 cm radial margin around the esophagus at the level of the primary tumor. In case of suspect lymph nodes, a CTVn (“nodal”) was created including all PET-positive or otherwise suspect lymph nodes with a short axis of above 1 cm. Organs at risk were spared as clinically appropriate. In case of definitive RCT a CTVb (“boost”) was defined for dose escalation to the primary tumor, with a reduced craniocaudal margin of 1 cm. To address a potential set-up error, planning target volumes (PTV) were defined with an additional isometric expansion of 0.5 cm.

GTV and CTV volumes were qualitatively analyzed by two radiation oncology experts and the DICE coefficient was calculated for quantitative analysis as a similarity measure ranging from 0 to 1, describing the overlap between two volumes. A high DICE coefficient therefore indicates a high similarity between the measured volumes. The standard-of-care contouring using CT and endoscopy (GTVp/n_CT) was considered the ground truth [14].

### Treatment and response assessment

Treatment plans were realized with either 6MV-photons from a Varian TrueBeam linear accelerator or 6MV-flattening filter free (FFF) photons using a Varian Halcyon linear accelerator. Sliding window IMRT or volumetric modulated arc therapy were performed with image guidance (regular cone-beam CT quality assurance). Patients treated in a neoadjuvant setting received 45 Gy in 25 daily fractions to the PTV. Patients in a definitive setting sequentially received twelve additional fractions as a boost to the initial tumor site to a cumulative dose of 66.6 Gy. Medical and physical treatment planning was performed with means of quality assurance according to the in-house standard procedure with multistep review before the initiation of treatment. Simultaneous chemotherapy consisted of weekly intravenous application of Paclitaxel 50 mg/m2 and Carboplatin AUC 2 (5 cycles in neoadjuvant treatment, 6 cycles in a definitive setting) [2, 15].

Four to six weeks after RCT, re-staging was performed (with five of ten patients receiving [68Ga]OncoFAP-PET/CT as part of response assessment). In case of a planned resection, re-staging was accompanied by endoscopic biopsy. Imaging results were correlated with pathological reports after tumor resection.

All statistical analysis was performed using IBM SPSS statistics Version 29.0 (provided by IBM, Armonk, Ny, USA). Non-normally distributed variables are expressed as median and range. Relative values are given in percentage. Wilcoxon signed-rank test was used to compare non-normally distributed paired variables. Unpaired, non-normally distributed data was compared with Mann Whitney U test. A value for *p* < 0.05 was regarded as significant.

## Results

### Patient demographics and data

Patients were evenly distributed between histological subtypes (50% adenocarcinoma, 50% SCC) with characteristics shown in Table 1.

**Table 1:** Patient characteristics

Number	1	2	3	4	5	6	7	8	9	10
Sex	W	M	W	W	M	W	M	M	M	M
Age [years]	80	65	83	64	59	69	81	70	68	63
Histology *A = Adeno-Ca* *S = SCC*	S	A	S	A	S	A	A	S	S	A
Grading	2	2	3	3	3	2	2	3	2	2
RCT-Setting *D = definitive* *P = preoperative*	D	P	P	P	P	P	D	D	P	D
cT stage	3	3	3	2	2	3	3	3	3	3
CT cN stage	2	2	1	1	0	0	0	0	0	1
EUS cN stage	+	+	+	+	+	+	-	-	-	+
FAPI cN stage	3	2	0	0	0	0	0	0	0	2
ypT stage	-	2	0	3	0	3	-	-	0	-
ypN stage	-	0	0	0	0	0	-	-	0	-
**[68Ga]OncoFAP** -PET Follow up	No	No	No	No	No	Yes	Yes	Yes	Yes	Yes

### [68Ga]OncoFAP-PET

All primary tumors were detected with [68Ga]OncoFAP-PET/CT. Primary tumors showed high uptake, with a median SUVmax of 19.4 (range: 10.8–28.8), and a median SUVpeak of 14.4 (range: 8.24–21.45). Elevated uptake was also seen in lymph nodes (SUVmax: median: 16.2 (range: 15.2–18.12), SUVpeak: median 10.1 (range: 9.5–11.3)). Uptake values of the primary tumor did not differ significantly (*p* = 0.35) between adenocarcinoma (median SUVmax: 15.9; range: 10.8–24.8) and SCC (median SUVmax: 23.0; range:14.8–28.8).

**Fig. 2:**
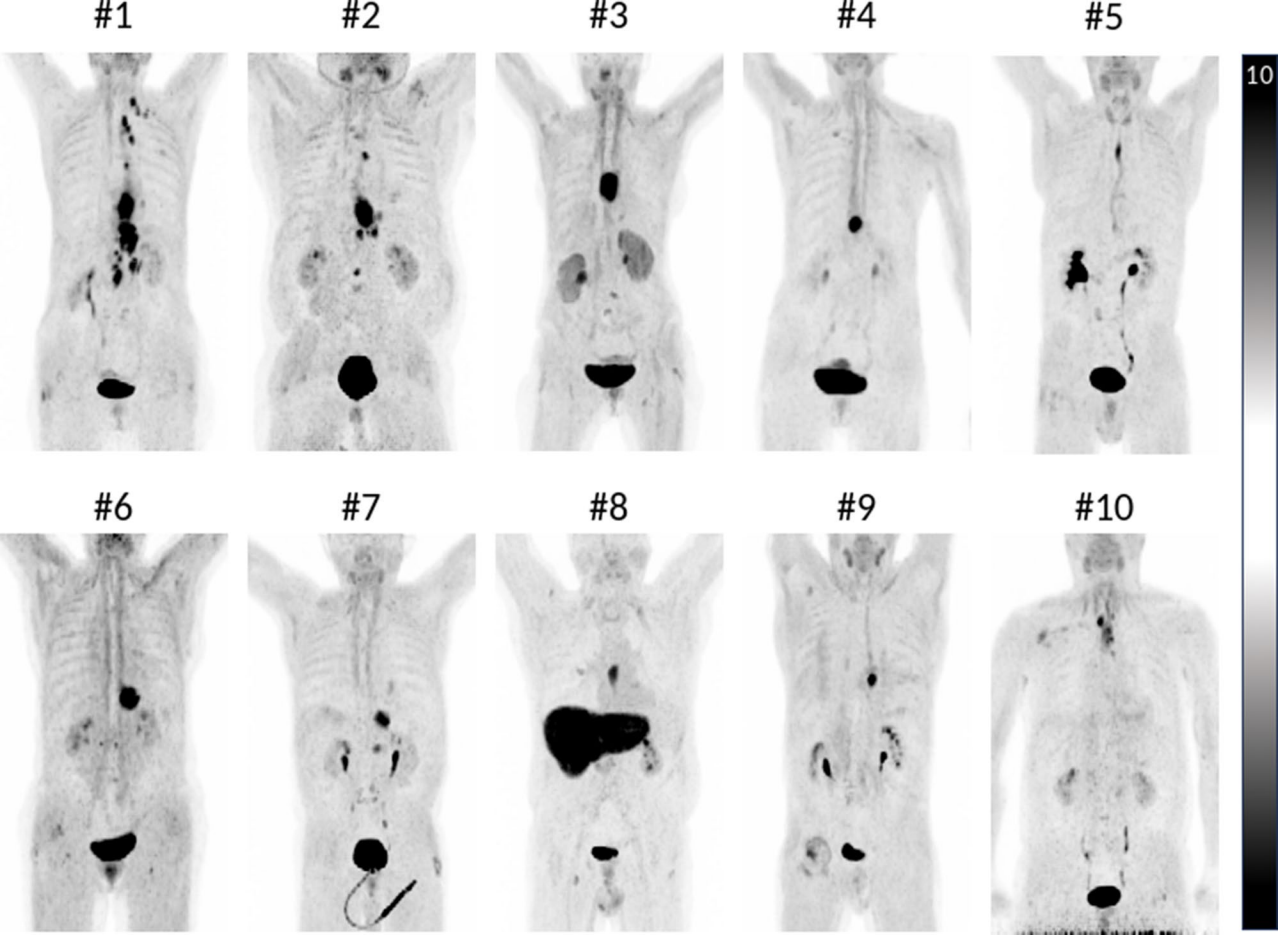
Maximum intensity projections of [68Ga]OncoFAP-PET of all esophageal cancer patients treated in our cohort

In 3/10 patients [68Ga]OncoFAP-PET changed TNM stage after initial standard EUS- and CT-based assessment by unmasking additional lymph node metastases (patients 1,2,3). While patients 1 and 2 were treated with definitive RCT, patient 3 received trimodal therapy and was staged ypN0 after neoadjuvant RCT. Figure 2 shows the projections of [68Ga]OncoFAP-uptake in the evaluated patients via maximum intensity projection.

**Fig. 3:**
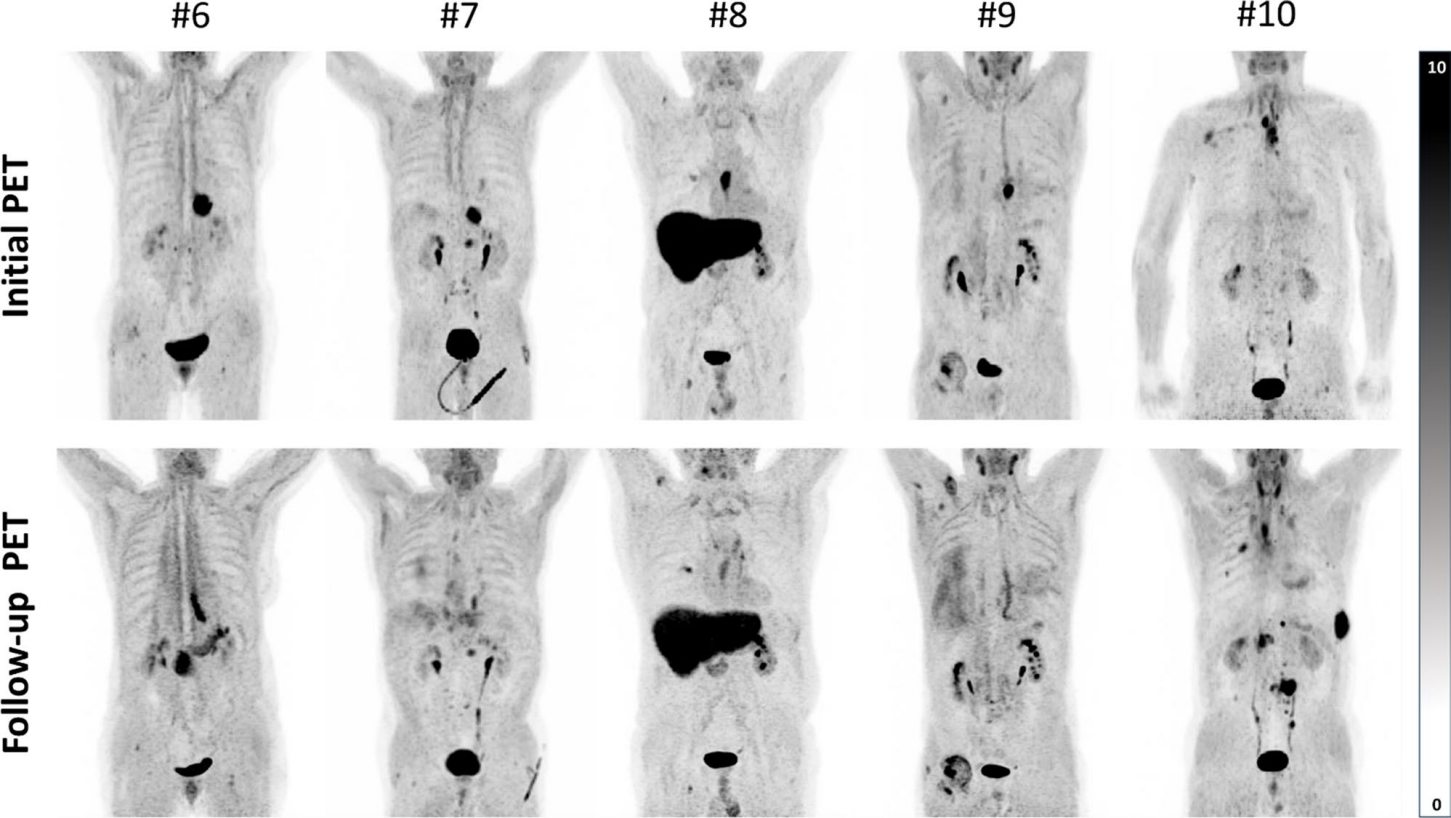
Comparison of [68Ga]OncoFAP uptake before and after RCT

To note that highly elevated uptake in the liver of patient 8 is related to a liver cirrhosis. Uptake in the liver remained unchanged in follow-up imaging (Fig. 3).

### Radiation therapy planning

The addition of [68Ga]OncoFAP-PET information in the process of target volume delineation led to an improved visibility of tumor infiltration as the extent of disease was visualized even in tumors directly adjacent to the heart or stomach. Setup-errors due to co-registration of PET/CT and planning-CT scans were reduced to a minimum by acquiring the PET/CT in the pre-defined treatment position.

[68Ga]OncoFAP-PET information led to a reduction in contoured GTVp volumes in 60% of cases. This was observed especially regarding the craniocaudal and gastric extent of the disease. An enlargement of the GTV was observed in 30% of cases with the inclusion of previously not suspected lymph nodes (patients 1,2 and 3). However, two patients with CT-graphically and/or endoscopically suspect lymph nodes did not show increased [68Ga]OncoFAP uptake in those nodes (patient 4 and 7). This would result in a potential omission of targeted lymphonodal irradiation. Nevertheless, all initially suspected manifestations were included in neoadjuvant RCT planning. The postoperative staging revealed a complete nodal response (ypN0) in both cases.

In several cases, the use of [68Ga]OncoFAP-PET-information led to a distinct remodeling of the GTV in different areas. These changes did not lead to a significant change in absolute volume but are represented in reduced DICE coefficients averaging 0.8. This underlines the substantial impact of [68Ga]OncoFAP-imaging on target volume definition in this cohort.

The volumetric changes and DICE coefficients are shown in Table 2.

**Table 2:** Tumor volume metrics and DICE coefficient values

Number	1	2	3	4	5	6	7	8	9	10
GTVp_CT [cm^3^]	65.6	62.3	55.8	23.0	13.4	53.9	36.6	16.4	11.8	36.1
GTVp_CT + PET [cm^3^]	64.2	62.3	56.2	20.4	11.1	55.4	39.1	10.9	8.5	35.1
Difference [cm^3^]	-1.4	0	+ 0.3	-2.6	-2.4	+ 1.5	+ 2.4	-5.5	-3.3	− 1.1
DICE	0.99	1	0.98	0.81	0.80	0.80	0.86	0.76	0.75	0.94
GTVn_CT [cm^3^]	37.7	5.0	4.2	-	-	2.0	-	-	-	8.3
GTVn_CT + PET [cm^3^]	38.5	8.8	0	-	-	0	-	-	-	9.4
Difference [cm^3^]	+ 0.8	+ 3.8	-4.2	-	-	-2.0	-	-	-	+ 1.1
DICE	0.76	0.73	-	-	-	-	-	-	-	0.94

### Treatment and response assessment

In our cohort, five patients received follow-up [68Ga]OncoFAP-PET with a mean of 5 weeks after finishing RCT as part of re-staging (Fig. 3). Of these patients, three were treated in a definitive RCT and two received trimodal therapy with PET/CT prior to resection. In all cases, SUVmax decreased significantly in post-therapeutic [68Ga]OncoFAP-PET compared to initial assessment (median decrease: 51%, range: 27–72%, *p* = 0.043).

However, a relevant residual uptake was observed in all patients (median SUVmax: 7.4, range: 5.2–10.8). Here, differentiation between residual tumor and reactive fibrotic changes after radiation therapy remains doubtful. Histopathological correlation is need to further elaborate on the specificity of uptake. In this study two patients with restaging [68Ga]OncoFAP-PET after neoadjuvant treatment, underwent surgery allowing for histopathological response assessment: Patient 9 had a lower residual uptake (SUVmax 6.1 and SUVpeak 4.0, compared to SUVmax 22.0 and SUVpeak 14.1 in initial imaging) and was confirmed a histopathological complete response, while patient 6 showed a higher residual [68Ga]OncoFAP uptake with lesser drop-off (SUVmax 10.8 and SUVpeak 8.0, compared to SUVmax 14.8 and SUVpeak 12.5 in initial imaging), revealing incomplete response in postoperative histopathology (10% viable tumor cells). Follow up imaging of [68Ga]OncoFAP-PET positive lymph nodes (CT: cN1, FAPI: cN2) after definitive RCT was obtained in one patient (patient #10) and showed a complete response of lymphatic manifestations, but revealed first diagnosis of multifocal metastatic spread to the bones, previously not detectable in any imaging. Comparison of pre- and post-treatment [68Ga]OncoFAP uptake is visualized in Fig. 3.

## Discussion

Imaging-based initial staging plays a key role for treatment decision-making in esophageal cancer. This may either lead to curative-intent neoadjuvant RCT followed by surgical resection or definitive RCT in a curative or palliative setting. Previous publications hint towards improved diagnostic sensitivity of FAPI-PET/CT compared to [18F]FDG-PET/CT [7]. In the ten patients evaluated in our study, [68Ga]OncoFAP-PET also demonstrated high uptake values with excellent visibility of tumor lesions in otherwise difficult locations (such as at the esophageal-gastric junction or in the retrocardiac mediastinum). [68Ga]OncoFAP is a relatively new FAPI-PET compound with favorable radiochemical properties, including low background activity and intense tumor uptake [16]. This tracer is therefore a powerful alternative to other FAP-compounds.

While the majority of previous studies on esophageal cancer predominantly included SCC [7, 17], our study included patients with an even distribution of histological subtypes. Uptake values were not significantly different between these two subgroups. This is in line with immunohistochemistry findings revealing comparable FAP expression in adenocarcinoma and SCC [18, 19]. This hints towards favorable diagnostic properties of FAPI-PET in esophageal cancer independent of subtype.

A well-known drawback of [18F]FDG-PET in esophageal cancer staging is the limited sensitivity for the detection of lymph node metastases [20]. The improved detection of lymph node metastases in FAPI-PET compared to conventional CT- and [18F]FDG-PET scans was shown in previous reports [7, 17]. Wegen et al. evaluated 32 patients with [18F]FDG and FAPI-PET dual-tracer PET imaging. The use of FAPI-PET led to the detection of two additional potential lymph node metastases which did not appear suspicious in [18F]FDG-PET alone [21]. Similar results were reported in our study, as FAPI-PET revealed additional suspicious lymph nodes in three out of ten patients.

Target volume definition in radiation therapy planning heavily relies on diagnostic imaging to accurately reflect the extent of disease. This is of utmost importance as an exact delineation of tumor sites impacts treatment effectiveness, as well as the occurrence and severity of side effects. In accordance with results previously published by Zhao et al., data from our study suggest that [68Ga]OncoFAP-PET has a significant influence on GTV definition compared to conventional imaging [8].

Thoracic OARs such as the lung and heart are in close proximity to the primary esophageal treatment volume and show a high potential for treatment-related toxicities. A correlation of lung dose distribution and volume with increasing risk of radiation pneumonitis and pulmonary fibrosis has previously been reported [22]. Heart tissue also shows relatively low tolerance to ionizing irradiation. Thresholds are low for late sequelae like pericarditis, arrhythmia and heart failure [23]. Therefore, maximal sparing of normal tissue by reducing treatment volumes is desirable, following the ALARA principle (as low as reasonably achievable). Defining the exact tumor extent in conventional CT-based treatment planning is difficult. This is compensated by a prolonged cranio-caudal extension of treatment volumes by several centimeters in modern contouring guidelines, resulting in relatively large target volumes. A more accurate depiction of tumor infiltration might enable smaller GTV-to-CTV margins, sparing healthy tissue in the esophagus and stomach. Limiting treatment-induced mucositis and dysphagia may reduce weight loss, shorten hospital stays, and mitigate the need for parenteral nutrition. PET-based de-escalation of RT treatment volumes has been successfully implemented in several types of cancer (e.g., lung cancer and lymphoma [22, 24]. The use of FAPI-PET as a basis for RT planning has also been explored in the context of gastrointestinal cancer treatment [25]. Still, a similar approach for esophageal cancer has yet to reach consensus. These results as well as our evaluation suggest that [68Ga]OncoFAP could be a very useful tool in the RT planning process in the future, with the aim of de-escalating treatment volumes.

Improved imaging capabilities may lead to better treatment decisions: In our cohort, one patient was changed from neoadjuvant to definitive RCT in a palliative setting because of the detection of distant malignant lymph nodes in FAPI-PET and to a lesser extend in CT after interdisciplinary decision making. Another patient was diagnosed with lower abdominal lymph node metastases, which were then included in GTVn during RCT and later resected. Preventing patients from unnecessary surgical interventions in situations of extended disease improves quality of life and is cost-effective. This has also been shown for [18F]FDG-PET. However, with the disadvantage of limited sensitivity for the detection of lymph node metastases [26].

The recently updated practice guideline for advanced tumors of the esophagus by the American Society of Thoracic Surgeons and the American Society for Radiation Oncology emphasized the increasing role of PET-based decision-making in upper gastrointestinal cancers in the form of response-assessment after induction chemotherapy [27]. The CALGB 80,803 trial [28] showed encouraging results regarding individualizing treatment with PET-response-adapted combined modality therapy for adenocarcinoma of the esophagus, leading to improved rates of pathological complete response after surgery. This approach might also be further explored regarding additional de-escalation of RT treatment strategies, for example in the form of response-adapted dose decisions for boost planning in patients with definitive RCT, or as a means of active surveillance after neoadjuvant treatment. However, these concepts need further prospective investigation. In any case, our experience highlights an emerging role of FAPI-PET in this context.

Additionally, PET-based assessment of treatment response was shown to be a predictor of survival in the CALGB 80,803 trial. For different FAPI-PET tracers, recent studies have also shown prognostic value of pre-treatment tracer-avid tumor volume in patients with esophageal cancer undergoing definitive RCT [9]. However, to our knowledge, our study is the first to report response assessment with FAPI-PET tracers after RCT in this setting. A reduction in uptake parameters is expected in response to RCT. This has already been reported for FAPI-PET based response assessment in esophageal patients undergoing chemotherapy alone [29]. However, one key disadvantage, regarding the limited specificity of FAPI-uptake needs to be acknowledged: FAP is overexpressed on cancer-associated fibroblasts, but also on activated fibroblasts in inflammatory disease, such as IgG4 related disease [30, 31]. RCT-related tumor cell death is associated with inflammation in and around the irradiated lesion and associated fibroblastic tissue response can lead to increased uptake, especially after ablative radiation doses, as recently pointed out by different authors [25, 32]. Larger studies of [18F]FDG-PET also demonstrated partly unspecific uptake after radiation therapy, not predicting histological response in multivariate analysis [33]. For FAPI-PET the optimal imaging time-point after radiation therapy still needs to be elaborated. Larger prospective studies are needed to provide information on the specificity of FAPI-PET uptake in the post RT-setting.

This study represents the experience of a single center. In addition, it is retrospective data from a small patient cohort. These are major limitations of this study. Prospective evaluation of larger patient collectives is needed to address the questions arising from our preliminary data that have not been answered yet, namely:

- the safety of reducing target volumes with FAPI-PET-based RCT.
- the role of FAPI-PET for response assessment during or after RCT is limited by unspecific FAPI uptake after RCT.
- data regarding predictive and prognostic parameters, e.g. in metastatic disease.

## Conclusions

This single-center experience hints towards an important role of [68Ga]OncoFAP for radiation treatment planning in esophageal cancer patients, in both SCC and adenocarcinoma. Target volume definition benefits substantially from the addition of FAPI-PET imaging. Future studies will define the role of FAPI-PET in treatment planning and should further elaborate on early response assessment in patients undergoing RCT.

## Data Availability

No datasets were generated or analysed during the current study.
